# What can the eyes tell us about atypical sexual preferences as a function of sex and age? Linking eye movements with child-related chronophilias

**DOI:** 10.1093/fsr/owad009

**Published:** 2023-03-23

**Authors:** Milena Vásquez-Amézquita, Juan David Leongómez, Alicia Salvador, Michael C Seto

**Affiliations:** Faculty of Psychology, Universidad El Bosque, Bogotá, Colombia; Department of Psychobiology, Laboratory of Social Cognitive Neuroscience, IDOCAL, University of Valencia, Valencia, Spain; Department of Social Sciences, Faculty of Human and Social Sciences, Universidad de la Costa, Barranquilla, Colombia; Faculty of Psychology, Universidad El Bosque, Bogotá, Colombia; Department of Psychobiology, Laboratory of Social Cognitive Neuroscience, IDOCAL, University of Valencia, Valencia, Spain; Forensic Research Unit, Royal Ottawa HealthCare Group, Ottawa, ON, Canada

**Keywords:** eye-tracking, visual attention, atypical sexual preferences, pedohebephilia, chronophilias

## Abstract

Visual attention plays a central role in current theories of sexual information processing and is key to informing the use of eye-tracking techniques in the study of typical sexual preferences and more recently, in the study of atypical preferences such as pedophilia (prepubescent children) and hebephilia (pubescent children). The aim of this theoretical-empirical review is to connect the concepts of a visual attention-based model of sexual arousal processing with eye movements as indicators of atypical sexual interests, to substantiate the use of eye-tracking as a useful indirect measure of sexual preferences according to sex and age of the stimuli. Implications for research are discussed in terms of recognizing the value, scope and limitations of eye-tracking in the study of pedophilia and other chronophilias in males and females, and the generation of new hypotheses using this type of indirect measure of human sexual response.

## Teaser

Eye tracking has recently been applied as a technique to measure indicators of atypical sexual interests such as pedohebephilia. This review seeks to outline the theoretical-empirical rationale to support the responsible use of the technique and to delineate its current impact and scope in understanding pedophilia, hebephilia, and other chronophilias.

## Introduction

The use of eye-tracking techniques has become popular in the last decade as a tool to examine cognitive processes underlying the processing of sexual information [1, 2]. This is because eye-tracking relies on the measurement of eye movements (fixations and saccades) as specific indicators of interest that provide relevant information about the underlying cognitive-emotional processing of sexual stimuli. This provides information beyond the direct genital measurements such as penile or vaginal plethysmography, or indirect ones obtained with autonomic physiological measurements such as pupillometry. Moreover, it has the advantage of circumventing general limitations such as the intrusiveness of genital physiological measurements or the effects of variations in the primary characteristics of the stimuli, such as illumination, on pupillometry techniques.

Eye-tracking is based on the recording of two types of eye movements, fixations (e.g. duration, number) and saccades (e.g. frequency, duration), which correspond to the measurement parameters or dependent variables most commonly used as indirect measures of sexual interests because of their relationship to visual attention toward sexually relevant stimuli. Eye-tracking allows us to know where and how observers look at the relevant sexual stimuli [3], for an example of an application to the study of sexual preferences, see [4].

Eye-tracking is a low-cost, non-invasive technique that is less susceptible to manipulation than techniques that rely on self-report or behavioral choices. It is therefore a promising methodology for the evaluation of both typical and atypical sexual preferences, compared to more invasive direct measures such as genital arousal assessment or social desirability-dependent measures such as self-report [5].

In contrast to other recent empirical reviews of eye-tracking research [2, 6], the aim of this review is to substantiate how current conceptual models of sexual information processing—mainly, the sexual information processing model developed by Spiering and Everaerd [7]—support the use of attentional paradigms that use eye-tracking as a useful indirect measure of typical sexual preferences such as androphilia (referring to teleiophilic androphilia or attraction to men) and gynephilia (teleiophilic gynephilia or attraction to women) and atypical preferences such as pedophilia (attraction to prepubescent children) and perhaps other chronophilias (age-based sexual preferences) [8]. These models include constructs—automatic and controlled processes, early and late attention, implicit and explicit memory, first fixation and duration of fixations—that help explain the relationship between sexual preferences and eye movements.

## Linking eye movements into the arousal information processing model

Eye-tracking assesses multiple cognitive processes, including perception, attention, memory, executive control and language [1, 2]. It is especially useful in the study of motivational processes in response to different types of incentives and potential rewards at the socio-emotional level. A well-conducted eye-tracking study allows solid inferences about preferred or prioritized stimuli. Likewise, eye-tracking makes it possible to measure variables that are difficult to measure through other methods, like the precise location of attention on an area of interest in both static and dynamic stimuli, and the cognitive resources invested at different moments of the presentation of a stimulus and according to the demand of the task. Finally, since the neural correlates of eye movements are well established, recording eye movements allows inferences about how the brain processes information [9].

Another advantage of eye-tracking over other direct or indirect measures is that it provides information about attention to specific regions of the body (e.g. face, chest, pelvis) that are key indicators to the observer of both the sex and age of the stimulus. Fixations to these specific areas reveal which stimuli are sexually relevant, and which parts of the body schema are of most interest, thus providing important information for identifying sexual preferences. Other indirect measures of visual attention (e.g. response time-based tasks) do not allow assessment of delayed attentional patterns on specific regions of the body. Its use in the evaluation of typical and atypical sexual preferences is, however, relatively new [10–14].

### Neurophysiological and cognitive basis of eye-tracking

Knowledge of the functioning of the visual system is important to understanding how eye movements are connected to visual perception and attention, as basic cognitive processes that are key to accessing more complex cognitive and affective processes. The human visual system makes fine distinctions in the focus of fixation through brief fixations on specific areas of interest. The fovea is the area of the retina responsible for this ability to perceive detail. Central foveal vision occurs due to corneal reflection that projects light onto the ocular fundus allowing fine inspection of 0–5° of the entire focus of visual input. About 90% of the time the eye remains in fixations, and only when the focus of attention changes do saccadic eye movements reposition the fovea toward the new focus of interest. The visual system works through connections between the retina and specific regions of the brain, the so-called visual pathways [1].

Eye trackers work from corneal reflection, allowing an infrared light to pass through the eye and be directed by the cornea to the retina, specifically the fovea. The corneal reflection is one of four images reflected on the four surfaces of the eye. These are called Purkinje images. The eye tracker works with image 1 and the pupillary center. A photosensitive camera receives the light signals reflected through the pupil and directs them to the central unit of the eye tracker, which algorithmically computes the relative position between the corneal reflection point and the pupillary center, allowing the fixation of the eye to be accurately determined. Being associated with the pupillary aperture, this same process is carried out in pupillometry techniques [15].

At the neurophysiological level, the structures and pathways involved in visual perception and attention are: the superior colliculi, important for the realization of eye movements; the primary visual area, responsible for the detection of stimuli; areas V2, V3 and V4, dedicated to the perception of movement and color; area V5, medial temporal and superior temporal area, process movement signals and control smooth eye movements; and the posterior parietal complex, involved in the realization of fixations. Likewise, there are two pathways responsible for visual perception and attention that connect with area V1, which are called dorsal and ventral. The ventral pathway is responsible for sensorimotor processing, i.e. the attentional *where* (localization and movements), while the dorsal pathway is responsible for cognitive processing, i.e. the attentional *what* [1].

Eye movements are not simple reflex actions; they are influenced both by bottom-up processes guided by external stimuli, and top–down processes involving higher order functions of the brain [1, 16]. Thus, the gaze is directed at places relevant to the organization of the action and achievement of individual goals [16]. Eye-tracking uses fixations and saccades as indirect measures of sexual preference. Fixations are intervals between saccades that bring stimuli of interest to the fovea, and saccades are ballistic changes in the position of the eyes, which allow moving from one position to another [3]. Fixations and saccades allow researchers to access the interaction between early and late visual attention processes, to be used as indicators of sexual arousal towards sexually preferred stimuli, according to the age and sex of the stimulus [11, 17].

The metrics used as indirect indicators of early and late attention to stimuli are the first fixations and the total duration and amount of fixations, respectively [11]. First fixations are relatively automatic, controlled by external stimuli; that is, they are addressed by the salient characteristics of relevant stimulus that competes for the attention of the subject with other stimuli when they are presented simultaneously. When two or more stimuli simultaneously appear in extrafoveal vision, they compete with each other. The measurement of early attention is only possible when, in the same scene, two or more stimuli compete for the observer’s cognitive resources. The most relevant stimulus will capture attention faster compared to the non-relevant stimuli. Hence, attention to a stimulus will depend on the stimulus with which it is competing [18]. The latency of first fixation, in the case of emotional stimuli within the first 500 ms [3, 18, 19], to one of these competing stimuli is a measure of early attentional capture by relevant characteristics of that stimulus [20, 21].

The total duration and number of fixations from when a stimulus appears until it disappears [18] (from 650 to 700 ms) [19, 22] is a parameter of late attention. Duration and number of total fixations are controlled because the person looks at what is most interesting to them, so this later attention is influenced by schemas, mood, and other cognitive factors. In this process, the relevant characteristics of the stimuli that coincide with the information stored in the explicit memory will maintain attention for a longer time and more frequently when they coincide with the observer’s preferences [23]. Fixations of 200–300 ms are sufficient for obtaining and processing conscious information of a stimulus or area of relevant interest. The total duration is usually related to the number of fixations on a stimulus or specific area of interest, and both are commonly taken as indicators of conscious processing and level of interest in relevant information [2].

### Information processing model of sexual arousal: the role of visual attention

Visual attention is the mechanism underlying the usefulness of eye-tracking in the study of sexual information processing. Visual attention involves two major types of processing: overt attention that involves eye movements and covert attention that occurs without eye movement. Eye-tracking serves the study of overt attention directed to relevant stimuli. The mechanisms that control visual attention are bottom-up and top-down. The former refers to the low-level characteristics of visual stimuli, such as brightness, luminance, color or motion, which indicate the salience of the stimuli. The latter relate to the observer’s goals, motivations, prior learning and moods [3]. It is here where the relationship between eye movements and sexual arousal becomes relevant.

It is possible to establish the correspondence between the mechanisms of visual attention (bottom-up and top-down) with the components of sexual arousal using the Information Processing Model of Sexual Arousal [7, 24]. Sexual arousal comprises subjective and physical sexual arousal. The former refers to the experience of sexual attraction and feelings (e.g. pleasure) toward sexual stimuli and erotic cues and awareness of physiological sexual arousal [25, 26], and the latter to physiological responses, which includes changes in cardiovascular, respiratory and genital tissues, including vaginal vasocongestion and lubrication in women, and penile erection in men [17, 27]. The interaction of both subjective and physical sexual arousal can lead to approach or avoidance of a sexual stimulus and sexual behavior [28].

A physiological response to a stimulus, such as pupil dilation to a visual stimulus, can occur very quickly, before conscious appraisal of that stimulus as sexual. However, even presentation of a sexual stimulus and physiological sexual arousal is not enough to trigger subjective sexual arousal. The variation in this experience will depend on the awareness and classification of the stimulus as sexually relevant to the observer [29]. A stimulus becomes sexually relevant from the interaction between cognitive and physiological processes that give rise to a particular subjective sexual response in the presence of the stimulus [7]. Given that genital or autonomic physiological techniques, such as pupillary dilation, are insufficient to access the subjective component of sexual arousal, eye-tracking offers important advantages, as it allows to approach these components, at least in principle.

In this paper, we link findings from eye-tracking research to the Information Processing Model of Sexual Arousal [7, 24] in order to conceptually support how the eye-tracking technique can be used to identify not only typical male and female sexual preferences, but also atypical preferences toward prepubertal children, and perhaps other chronophilias. This model has been the basis for the further development of more recent models applied to explaining sexual violence, such as the Incentive Motivation Model developed by Toates [30]. We selected the Information Processing Model because it highlights the importance of pre-conscious and conscious attention following the presentation of a sexual stimulus as an indicator of sexual interest.

The identification of aversive and appetitive stimuli is essential for survival and reproduction, and this is a function of attention [31]. Sexual stimuli are appetitive stimuli that offer information about the sexual maturity, reproductive potential, fecundity, healthiness, and genetic quality, perceived as the sexual attractiveness of a potential partner, so that attention has a relevant role to obtain that information through the perceptible signals of sexual stimuli [32–36].

The Information Processing Model of Sexual Arousal [7, 37] explains the interaction between the physiological and subjective mechanisms that give rise to the sexual response, based on the integration of bottom-up (automatic) and top-down (controlled) processes [17, 38]. The connecting bridge between these processes and sexual arousal is attention, and eye movements are a manifestation of the cognitive mechanisms that underlie this link.

Typically, when a sexual stimulus in front of the observer signals possible sexual reward, the conspicuous cues (e.g. prominent breasts in women, or broad shoulders in men) generate a pre-conscious orientation towards the stimulus. This is because of the activation of implicit memory, which stores relevant information about the stimulus that is related to innate reflexes and/or conditioned responses [7], as well as the person’s history of sexual reward, based on past experiences and learning [30, 39]. Non-conscious initial physiological responses occur if stimulus features activate implicit memory, resulting in genital sexual arousal [7, 40].

Awareness of genital arousal leads to the activation of a controlled mechanism of conscious attention to the specific features of the stimulus. If the stimulus corresponds to the content stored in the explicit memory in the form of sexual scripts, memories, attitudes, fantasies and expectations of reward or cost, they induce a cognitive evaluation that can result in the experience of subjective sexual arousal [24], in which a closer approach or contact with the sexually desired stimulus might be sought [41]. Subjective sexual arousal can lead to greater physiological sexual arousal through a positive feedback loop. More suitable sexual stimulation will result in greater sexual arousal, which in turn increases the likelihood of engaging in behavior [42]. If approaching the stimulus is reinforced, future excitation will increase and future inhibition will decrease [41]. There is also a goal-oriented executive control that moderates the approach behavior towards the stimulus [30]. Thus, a complete sexual response integrates mechanisms involving attention, memory and self-regulation [7, 37].

The pre-conscious and conscious attention processes that are activated by a sexually preferred stimulus can function as indirect indicators of sexual arousal. Early or pre-conscious attention is related to genital response, while late or conscious attention is related to subjective sexual arousal [43, 44]. Thus, the attentional patterns can function as predictors of excitation patterns according to the stimuli (e.g. sex and age) and according to the characteristics of the observer (e.g. sex, sexual preferences).

The Information Processing Model continues to be the main basis for research on both typical [45–48] and atypical sexual responses [14, 49, 50]. The model has been supported through neurobiological evidence [51, 52], and has been integrated into the most recent explanatory models of sexual violence, such as the Incentive-Motivation and Hierarchical Control Model [41]. This model integrates pre-conscious and conscious components of sexual stimulus processing, from its early phases, in which motivational processes triggered by incentives and/or cognitive representations of incentives are involved, to its late phases, in which a slow conscious executive control is involved, which allows delaying gratification and regulating behavior [30].

**Figure 1:**
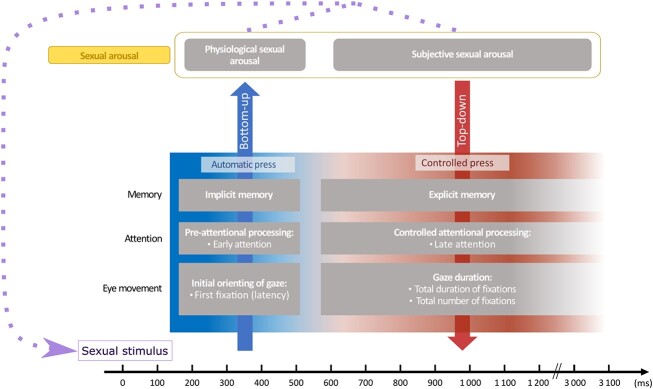
Integrative model of eye movements in the sexual arousal information processing system. The horizontal blocks represent the cognitive processes underlying the eye movements towards sexually relevant stimuli that give rise to the sexual arousal. The horizontal panels represent: in blue, the automatic bottom-up processes, which give rise to the beginning of the physiological sexual arousal; and in red, the controlled top-down processes, which give rise to the subjective sexual arousal. The *x*-axis represents a timeline of the hypothetical course of automatic and controlled processes, from the appearance of the stimulus, through the beginning of the pre-attention process, advancing to the controlled processing of sexual information. The purple arrow represents the cycle of the complete sexual response, which occurs when automatic and controlled processes interact, increasing physiological arousal and increasing attention on the visual stimulus or relevant regions that maintains the expectation of reward

We depict this relationship between eye movements and the sexual arousal in an illustrative model (see Figure 1). The figure describes the correspondence between the components of sexual arousal, the underlying pre-conscious and conscious attentional processes, also called early and delayed attention, and the eye movements elicited by sexually relevant stimuli. Applying the concepts of this illustration to pedophilic sexual interests, when a sexually relevant stimulus competes for the observer’s attention with other stimulus irrelevant to the observer (e.g. child stimulus *vs.* adult stimulus), if the characteristics of the relevant stimulus match information stored in implicit memory (e.g. association of the child body with reduced discomfort or increased sexual arousal) pre-attentional bottom-up mechanisms are activated leading to an initial gaze orientation or first fixation to the stimulus with salient global features (e.g. child body shape) and genital physiological arousal occurs. Subsequently, if the global or specific features of the relevant stimulus (e.g. immature genitals) match the content of explicit memory (e.g. distorted beliefs about children and sex), conscious top-down attentional mechanisms are activated leading to overt attention and exploration of specific areas that have a cognitive representation with sexual significance (e.g. breast, pelvis, face); this conscious attention translates into an increase in the total number and duration of fixations on the relevant stimulus during the total time of its presentation [14].

## Eye-tracking and atypical sexual preferences: empirical evidence

Without conducting a systematic review, we identified relevant empirical evidence of the use of eye-tracking in the study of atypical sexual preferences according to sex [10, 53–55] and age of the stimuli [49, 54, 56, 57]. These studies were included because they use the eye-tracking technique (measurement of fixations and saccades) for sexual research and use whole-body visual stimuli. They present original research results—in the case of studies based on sex-atypical sexual preferences—include samples of one or both sexes with same-sex and other-sex sexual preferences, and present male and female body stimuli—in the case of studies on pedophilic sexual preferences—including samples of child sexual offenders with confirmed or presumptive pedophilic sexual interests, using stimuli from real or non-real adult and child bodies.

### Atypical sexual preferences according to sex of stimuli: gynephilia and androphilia

Sexual preferences according to the sex of stimuli within the gynephilic (attraction to women)–androphilic (attraction to men) [43, 58, 59] continuum can be typical or atypical. Sexual preferences for women by men and for men by women are species-typical, whereas same-sex preferences are atypical, involving a minority of the population [60, 61]. Typicality as we undrestand it here refers only to statistical rarity. Same-sex sexual preferences are non-paraphilic atypical sexual preferences [62] because they are not sexual disorders and do not include paraphilic preferences that involve sexually immature persons, objects, or unusual activities (e.g. sex or sexual intrusive and persistent fantasies by coercive sex or sex with children) [63].

Regarding sexual preferences for sex, extensive empirical evidence has shown that gynephilic or androphilic men without paraphilic sexual interests show a specific sexual response pattern to women or men, respectively, and this manifests in both the early and late attentional patterns [10, 45, 54, 64]. This has been shown using different methods, such as genital measures [65–68], subjective self-report [10, 65, 69, 70], and attentional paradigms [71–73], including eye-tracking [10, 53–55].

In women, the response pattern is different. Early studies revealed that women, regardless of their gynephilic or androphilic preferences, showed a similar genital response pattern towards stimuli depicting both men and women, which has been described as a non-category-specific sexual arousal pattern [55, 67, 70, 74, 75]. A non-specific sexual response is defined as a pattern of similar sexual response to both sexually preferred and non-preferred stimuli, in which there is lower congruence between physiological and subjective sexual arousal [68]. More recently, studies have found that only exclusively androphilic women showed a non-specific genital response to their self-reported preferred sex, inconsistent with their self-reported sexual arousal [66, 76, 77], while women reporting any gynephilia showed a category-specific sexual response, similar to that observed in men [66, 78].

These findings have been consistent with studies that use eye-tracking, in which it has been found that the early attention pattern of men tends to be gender-specific, while the pattern of women tends to be non-specific. This can be contrasted to the late attention pattern that has been found to be gender-specific in gynephilic men and androphilic women, although with weaker effects in women [44, 54, 79].

These findings indicate that sexual preferences can be explored through the visual attention patterns to sexually relevant stimuli, since some aspects of visual attention seem to be guided by the observer’s sexual interests. Thus, the attentional processing of sexual stimuli may vary depending on the stage at which the information is processed (early or late) and thus provide information on the underlying mechanisms (automatic or controlled) of deviant atypical sexual preferences, such as pedophilia.

### Atypical sexual preferences according to age of stimuli: chronophilias

It has recently been proposed that sexual preferences can also be categorized according to age, or *chronophilias* [8]. Chronophilia categories include nepiophilia (babies and children up to two years old), pedophilia (prepubescent, typically between 3 and 10 years old), hebephilia (pubescent, typically between 11 and 14 years old), ephebophilia (teenagers, typically between 15 and 17 years old), teleiophilia (sexually mature young adults, typically between 18 and 30 years old), mesophilia (middle-aged adults, between 31 and 59) and gerontophilia (elderly adults, typically over 60 years old) [8]. Teleiophilia is the species-typical sexual preference for sexually mature adults.

**Figure 2:**
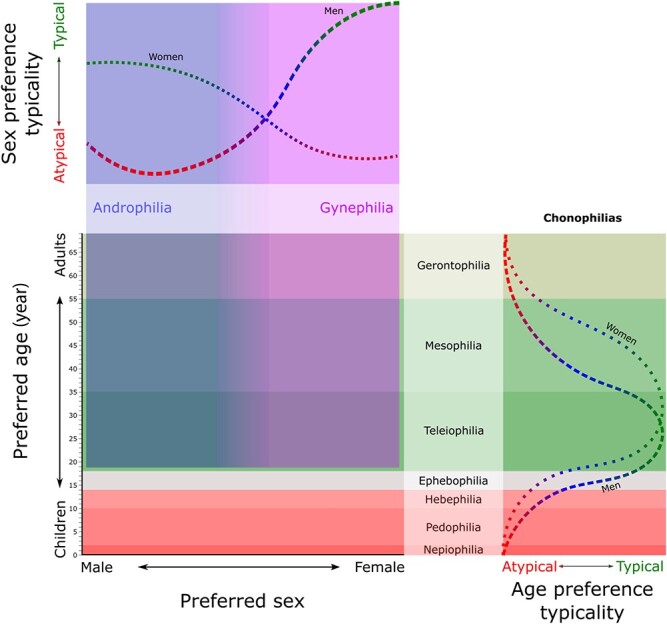
Hypothetical model of the variations in the typical and atypical sexual preferences according to the age and sex of the preferred stimulus and representation of the distribution of the typicality according to age and sex of the stimuli, estimated from indirect evidence [8, 65, 77, 81, 83]. In typicality according to the age of the stimulus, paraphilic chronophilias are represented in red, and in green tones those that are not considered paraphilias. In typicality according to the sex of the stimulus, the bands, blue (androphilia) and purple (gynephilia), represent sexual preferences for sexually mature men and women

We propose a hypothetical model of typical and atypical sexual preferences that integrates both the age and sex dimensions of the stimuli (Figure 2). The distribution along the typical–atypical continuum according to the age and sex of the stimuli is hypothetical, estimated, and constitutes a starting point for research. The typicality according to the age of the stimulus, is estimated from what is known about the frequency of chronophilias in men, given the indirect evidence of mainly teleiophilic preferences and relatively few cases of pedohebephilia among women (for review, see [8]). Seto [8] speculated that men would show a bias towards younger age categories, whereas women would show a bias towards older age categories, given normative data on age preferences in dating and marriage preferences and behavior, and reflecting sex differences in human reproductive strategies [80]. Likewise, for the typicality according to the sex of the stimulus, indirect evidence suggests that the frequency of non-exclusively androphilic women may be higher than that of non-exclusively gynephilic men, taking into account the specificity of men’s sexual response and the non-specificity of androphilic women’s sexual response [65, 77, 81].

The available evidence using eye-tracking, and even any other direct or indirect measure of sexual arousal, is limited to the distinction of sexual preferences for prepubescent and pubescent children (Tanner Stages 1–3), postpubescent adolescents (Tanner Stage 4) and adults (Tanner Stage 5) to study pedohebephilic atypical sexual preferences [49, 54, 56, 57]. However, there is as yet no evidence using eye-tracking or other objective assessment methods for mesophilia or gerontophilia.

#### Category-specific sexual response: gender differences

We summarize the evidence on category-specific sexual response in this section for two reasons. First, to highlight that most of the information available about these atypical sexual preferences such as pedophilia and hebephilia comes from research in men; it is clear that atypical age preferences are more common in men than in women [8]. And second, paradigms based on the visualization of stimuli using eye-tracking have been consistent in showing a low sensitivity to detect effects, especially those of sex on visual attention, and are therefore possibly insufficient to identify typical and atypical sexual preferences in women, especially exclusively androphilic women [13, 26, 45, 54].

The evidence of category-specific sexual response among men supports the use of direct and/or indirect measurements of sexual interest as indicators of specific sexual preferences according to the sex and age of the stimuli, proving useful in cases in which men deny paraphilic sexual preferences [14, 54, 70].

Given non-specificity of the female sexual response according to the sex of the stimulus for exclusively androphilic women, and the very low prevalence of pedophilia among women [81, 82], it is unclear if direct or indirect sexual interest measures to assess the sexual preferences of women according to the age of the stimulus will work, though gynephilic and androphilic women without paraphilic sexual interests show an attentional pattern consistent with their subjective sexual arousal towards sexually mature adults [11, 54, 57, 71, 83].

#### Atypical age sexual preferences and eye-tracking

Although research with gynephilic and androphilic men has shown that there is a pattern of specific early and late attention to adult stimuli of the preferred sex [11, 54, 84], only three laboratory groups in Germany [49, 85, 86], the United Kingdom [87] and Colombia [14] have used eye-tracking as a measure of pedophilic sexual interest with forensic samples.

Results have been mixed, initially showing that men with pedophilic sexual interests show early and late attention bias towards children, compared to men without sexual offenses involving children or men in the general population without pedophilic sexual interests or sexual offenses involving children [86]. However, more recent studies have replicated only the early attention bias, not the late attention results [14, 49]. Two studies have observed greater late attention to the chest [14, 87], rather than the pelvic region as observed by Fromberger et al. [49]. Recently, there has been experimentation with eye-tracking and implicit viewing paradigms using sexual stimuli as distractors, to look for evidence of top-down control problems in the child stimuli viewing of pedophilic compared to non-pedophilic men [12, 50, 85]. Jordan et al. [85] found that pedophilic men show less attention control when the sexual stimulus is a child distractor, compared to two non-pedophilic control groups (men who had non-sexually offended and nonoffending community men), evidencing faster first fixations and longer duration of fixations for child distractors. This study showed good levels of discrimination between pedophilic and non-pedophilic men. However, it showed that in this type of paradigm, task complexity could matter, motivation and task performance should be assessed, and it could be important to include non-sexual control distractors. In a second study, Jordan et al. [12] compared 11 nonforensic outpatient and 22 forensic inpatient men who were pedophilic. However, this time they found no significant differences between the groups in attentional control.

A more comprehensive review of the results of research using eye-tracking to analyze pedophilic interest can be found in Godet and Niveau [88], who identified only six studies with this objective, including those mentioned above. The authors conclude that although the results suggest that eye-tracking is useful for discriminating between men with pedophilic sexual interests from non-pedophiles, there is limited research on this topic, with small and diverse samples, and more clarity is needed on its relevance in the forensic context.

Finally, Supplementary Table S1 provides a classification of the indirect measures, including eye-tracking techniques applied to the assessment of sexual arousal and sexual preferences according to the sex and age of the stimuli, as well as the empirical literature available.

## Limitations of eye-tracking in atypical sexual preferences research

All methods for the evaluation of atypical sexual preferences include strengths and limitations, including eye-tracking paradigms [6]. First, experimental paradigms using indirect measures, such as eye-tracking, although based on a solid conceptual framework, ignore some of its key components, like the value of triggering incentives. Studies with experimental stimulus visualization paradigms should include, in their analysis of response to sexually atypical stimuli, the history of incentives. In the case of pedophilia, this could include asking about self-reported intensity of sexual attraction to prepubertal children, and if tenable, reports of their use of sexually explicit content depicting children and prior sexual contacts with children.

Second, most studies use stimuli without sufficient validation to show that they can encompass idiosyncratic preferences of those evaluated. For someone who is sexually attracted to young men, for example, their response may be affected by the muscularity of the depicted individuals, with some more attracted to highly muscularized men and others attracted to non-muscular men. Other characteristics that could influence sexual responses include height, hair color, and skin tone. Therefore, the selection of stimuli is key in designing an experimental paradigm to measure sexual preferences according to the age of the stimuli [14, 49, 87]. Difficulties in stimulus validation, specifically in the assessment of pedophilic sexual interests, also involve the ethical and country-specific limitations of using nude depictions of real children, so virtual or non-real stimuli have been used [49, 86, 89], but the sensitivity of discriminating pedophilic sexual interests using these stimuli compared to real stimuli, and how much this might affect the validity of the stimuli, is unknown. Studies comparing this effect would be valuable.

And third, studies ought to be replicated and expand the research to establish the psychometric properties of test–retest reliability, as well as validity, of eye-tracking as a measure of typical and atypical sexual preferences [86]. The design of experimental paradigms of parallel forms is necessary to measure test–retest reliability, which would shed light on the stability of sexual interests assessed using eye-tracking. Studying the validity of the attribute categories in relation to the type of stimuli will identify critical stimulus features, including what cues are most salient, what cues signify sexual content, and what contextual cues influence responding. Validity studies are necessary to define the usefulness of different types of experimental paradigms; for example, those using free visualization *versus* those using forced choice, which are distinguished from each other, since the former use the genuine gaze pattern as an indicator through the instruction ‘look freely at the pictures as you normally would’, and the latter guide the visual attention pattern through a restricted instruction, for example ‘which of the two stimuli seemed more attractive to you’. The latter have been criticized [90] for biasing the gaze pattern toward the target stimuli, so comparative evidence between the two types of experimental paradigms is needed.

Discriminant validity studies are also suggested to determine whether eye-tracking effectively captures attentional biases toward sexually relevant stimuli compared to other irrelevant sexual stimuli, and further convergent validity studies to determine the correlation of eye-tracking measures with other measures of sexual interest (e.g. genitalia or self-reports).

The latter have been questioned due to the possibility of involving delay processes based on the evaluation of characteristics of the stimulus, which can be faster to discard stimuli that do not belong to a category (i.e. stimuli evaluated as non-sexual [91]), compared to paradigms visualization that does not involve restricting the natural response to the stimulus.

A strategy to increase the reliability and validity of eye-tracking is triangulation with other direct and indirect methods that offer information about typical and atypical sexual preferences, and that have reported good psychometric properties such as the Revised Screening Scale for Pedophilic Interests (for men who have sexually offended against children) [8] or the Implicit Association Test (IAT) or viewing time measures [92, 93].

A relevant technical aspect in the use of eye-tracking to reliably measure typical and atypical sexual preferences, has been the lack of consistency in the upper limit latency for first fixations. That is, how long can we still consider a first fixation to indicate early attention; this leads to inconsistencies in the findings regarding this variable as a measure of early attention towards stimuli of sexual relevance [14, 49, 86]. This is important, since, for example, Imhoff et al. [83] showed that the psychometric properties of visualization times during the evaluation of the sexual attractiveness of a sexually preferred stimulus were better when stimuli were presented in less than a second, which demands a rapid response. This is important because the time restrictions and the demand for quick responses can be better indicators of automatic sexual responses towards sexually preferred stimuli, so the duration of the stimulus and the task are very important to increase the validity of an experimental paradigm.

A new experimental attentional paradigm using eye-tracking has recently been proposed to measure non-conscious cognitive processes that underlie the processing of sexual information in early stages [94], attempting to overcome the limitations of free-viewing and forced choice paradigms, which report evidence about uncontrolled processes from latencies of the initial fixation on the sexually relevant stimulus. Experimental tasks such as cued pro- and anti-saccade paradigms that measure saccades (a rapid movement of the eye between fixation points) approaching the sexually relevant or irrelevant stimulus (prosaccades), and saccades moving away from the sexually relevant or irrelevant stimulus (antisaccades), have shown potential as an indirect measure to explore initial attentional processes based on sexual interest [94].

This paradigm has an advantage in the validity and reliability of the task to measure early attentional processes. Compared with previous studies using the first fixations [14, 49], this task using sexual stimuli is based on the saccades that occur before the first fixation and that offer more precise information on earlier stages of the localization of attention, in addition to help understanding the conflict between automatic and controlled processes in the generation of a saccade [94, 95]. Evidence of its usefulness has been proven in studies on attentional biases towards emotional stimuli in clinical disorders [96–99]. It has been tested with sexual stimuli in a recent study [94], and a recently published study used this paradigm in assessing the impact of a single session of transcranial stimulation on the response biases of pedophilic compared to non-pedophilic men [100].

Finally, another significant limitation is that the most studies are concentrated in non-clinical groups with typical sexual preferences [10, 64], with very limited evidence in clinical and forensic samples with paraphilic atypical sexual preferences. It is hoped this review spurs interest in conducting community research on atypical sexual preferences, including paraphilias.

## Future directions and conclusions

The literature reviewed reveals important contributions of eye-tracking during the last decade to the understanding of the cognitive mechanisms underlying typical and atypical sexual preferences, but it also shows the obstacles (e.g. insufficient evidence at the moment with clinical and forensic samples; lack of precision on psychometric properties of experimental sexual stimuli visualization paradigms) for concluding its usefulness in this field.

Despite this, eye-tracking is less expensive and invasive than genital measures in sexuality studies and is more accessible because it does not require specialized labs and technicians to conduct. It may also be more accessible to clinical and research use, especially for stigmatized interests such as pedophilia and in clinical or forensic contexts [101, 102].

Eye-tracking has become a key method to test the theories of sexual arousal that give a central role to attention, and the interaction between automatic and controlled cognitive processes that give rise to the human sexual response [17, 24, 30]. This, in addition to being an alternative technique to self-report (given how easy it is to lie or manipulate what is reported) has been useful for testing cognitive theories about pedophilia [103], which share the common notion that there are underlying cognitive mechanisms that could lead to attentional biases towards atypical sexual stimuli (e.g. prepubescent, and/or pubescent children). It can even be useful for testing newer integrative models, both for understanding sexual preferences according to sex and according to age, including the entire spectrum of chronophilias [41, 104].

The idea of an early attention bias towards child stimuli suggests the existence of mechanisms related to implicit memory, in which atypical non-conscious associations can occur between concepts related to sex and the sexually immature bodies of children. This could offer gratification or reduce discomfort, reinforcing attention biases towards children as an object of desire, and mediating the motivation to approach or not, which would eventually lead to the decision to move from fantasy to the search for sexual contact with a child [41].

Regarding late attention processes, the evidence about pedophilia is inconclusive. The measurement of this process is much more susceptible to the conscious control of the subject, leading to inconsistencies in the investigation of differences between pedophilic and non-pedophilic men in the amount of conscious attention to child stimuli [49]. However, eye-tracking has allowed the identification of a differential pattern of attention to specific regions of the sexually immature body such as the chest [14, 84] and pelvis [49]. This is information that is not provided by other indirect measures and highlights the importance of replicating and refining attention paradigms using eye-tracking.

Eye-tracking research should continue, with larger general samples, clinical and non-forensic samples with pedophilic sexual interests, and forensic samples that differentiate those who have sexually offended into groups with and without pedophilic sexual interests, since not all who have sexually offended are pedophilic and not all pedophilic individuals have sexually offended [105, 106]. Additionally, the full spectrum of chronophilias should be included in research of sexual preferences according to age, just as the full spectrum of the gynephilia-androphilia continuum has been included in the study of sexual preferences according to sex.

Eye-tracking studies differentiating pedophilic from non-pedophilic child sex offenders, and pedophilic men who have offended from those who have not, could have clinical and forensic applications because eye-tracking, as the technology becomes more affordable, could be useful in the clinical assessment of pedophilic sexual interests when more intrusive measures such as phallometric assessment of penile responses is not available or would be refused. Because eye-tracking can distinguish automatic from controlled attention, it could be used to monitor the impact of cognitive-behavioral treatments designed to increase self-regulation of sexual arousal on controlled attention specifically. Longitudinal follow-up research could determine if eye-tracking results can predict sexual recidivism, as phallometric testing and visual reaction time measures can [106].

Likewise, a new line of investigation is possible in forensic samples of women who have sexually offended against children and who may have pedophilic sexual interests. Eye-tracking can help in exploring these new hypotheses since nonoffending women have shown age-specific sexual interest in eye-tracking [54] and other indirect measures [56, 71, 73] for sexually mature adults. Indirect measures, including eye-tracking, may be promising in assessing atypical sexual preferences in women [107].

Although we are far from being able to use eye-tracking and attentional paradigms as clinical diagnostic tools, research is the main way to find its usefulness and reliability as an indirect measure of sexual preferences, and as a potential complementary tool in clinical and forensic settings. This work is an invitation to continue doing research using this technique and to extend its application range to the clinical-forensic field, supported by solid theoretical models on sexual information processing as well as typical and atypical sexual preferences according to age and sex.

## Supplementary Material

Supplementary_Materials_owad009
