# Supplementary material for: What can the eyes tell us about atypical sexual preferences as a function of sex and age? Linking eye movements with child-related chronophilias
Source: Forensic Sci Res. 2023 Mar 23;8(1):5–15. doi: 10.1093/fsr/owad009 (PMC10498142; doi:10.1093/fsr/owad009)
Supplement: Supplementary_Materials_owad009 [file supplementary_materials_owad009.docx]

**Supplementary Materials**

| *Table S1*. Classification of the main indirect methods used in the evaluation of sexual arousal and sexual preferences according to the sex and age of the stimuli. Main limitations and outstanding literature in each case are included. | | | | |
| --- | --- | --- | --- | --- |
| Measurements | Types | Subtypes | Outstanding reference literature | |
|  |  |  | According to sex | According to age |
| Main limitations:   - Measures attention, but not sexual arousal - Complex and expensive apparatus (mainly in central measures) - Lack of standardization of some procedures - Discriminant validity is still not equal to or surpasses direct genital measures   (Carvalho et al., 2020; Kukkonen et al., 2007) | **Periferical-somatic** | Thermography | (Chivers et al., 2010)  (Huberman & Chivers, 2015)  (Kukkonen et al., 2007)  (Kukkonen et al., 2010)  (Tavares et al., 2018) | Unavailable |
|  |  | Pupillometry | (Rieger & Savin-Williams, 2012)  (Rieger et al., 2015)  (Snowden et al., 2019) | (Bouchard et al., 2019)  (Attard-Johnson et al., 2016)  (Attard-Johnson et al., 2017)  (Caoilte Ó Ciardha et al., 2018) |
|  |  | Eye tracking | (Jones, 2013)  (Vásquez-Amézquita et al., 2019)  (Fromberger, Jordan, von Herder, et al., 2012)  (Wenzlaff et al., 2016) | (Vásquez-Amézquita et al., 2019)  (Fromberger, Jordan, Steinkrauss, et al., 2012)  (Jordan et al., 2016)  (Fromberger et al., 2013) |
|  | **Central** | Neuroimaging | (Ponseti et al., 2007)  (Hu et al., 2011)  (Mitricheva et al., 2019)  (Strahler et al., 2019)  (Borg et al., 2014)  (Poeppl et al., 2016) | (Ponseti et al., 2018) (Ponseti, 2012a)  (Ponseti, 2012b)  (Jordan et al., 2015)  (Poeppl et al., 2013) |
|  | **Behavioral-Instrumental**   - Based on response *latency or choice reaction* time | Explicit visualization:     - Implicit Association Test (IAT) - Rapid Serial Visual Presentation (RSVP) - Choice Reaction Time   Implicit visualization:   - Implicit Relational Assessment Paradigm (IRAP) | (Snowden & Gray, 2013)  (Zappalà et al., 2013)  (Rönspies et al., 2015)  (Rönspies et al., 2015) | (Hempel et al., 2013)  (Babchishin et al., 2013)  (Gray et al., 2005)  (Crooks et al., 2009)  (Dawson et al., 2009)  (Zappalà et al., 2016)  (Dombert et al., 2015)  (Gress & Laws, 2009)  (Flak et al., 2009) |
|  |  | - Modified Stroop Test | Unavailable | (C. Ó Ciardha & Gormley, 2012)  (Smith & Waterman, 2003)  (Bourke & Gormley, 2012)  (Spada & Jeglic, 2016) |
|  | - Based on *viewing time* | - Viewing time | (Ebsworth & Lalumière, 2012)  (Imhoff et al., 2010)  (Fromberger et al., 2015)  (Rullo et al., 2010) | (Babchishin et al., 2014)  (Mokros et al., 2013) (Harris et al., 1996)  (Bourke & Gormley, 2012) |

**References**

Attard-Johnson, J., Bindemann, M., & Ó Ciardha, C. (2016). Pupillary Response as an Age-Specific Measure of Sexual Interest. *Archives of Sexual Behavior*, *45*(4), 855–870. https://doi.org/10.1007/s10508-015-0681-3

Attard-Johnson, J., Bindemann, M., & Ó Ciardha, C. (2017). Heterosexual, Homosexual, and Bisexual Men’s Pupillary Responses to Persons at Different Stages of Sexual Development. *The Journal of Sex Research*, *54*(9), 1085–1096. https://doi.org/10.1080/00224499.2016.1241857

Babchishin, K. M., Nunes, K. L., & Hermann, C. A. (2013). The Validity of Implicit Association Test (IAT) Measures of Sexual Attraction to Children: A Meta-Analysis. *Archives of Sexual Behavior*, *42*(3), 487–499. https://doi.org/10.1007/s10508-012-0022-8

Borg, C., de Jong, P. J., & Georgiadis, J. R. (2014). Subcortical BOLD responses during visual sexual stimulation vary as a function of implicit porn associations in women. *Social Cognitive and Affective Neuroscience*, *9*(2), 158–166. https://doi.org/10.1093/scan/nss117

Bouchard, K. N., Moulden, H. M., & Lalumière, M. L. (2019). Assessing Paraphilic Interests Among Women Who Sexually Offend. *Current Psychiatry Reports*, *21*(12), 121. https://doi.org/10.1007/s11920-019-1112-2

Bourke, A. B., & Gormley, M. J. (2012). Comparing a Pictorial Stroop Task to Viewing Time Measures of Sexual Interest. *Sexual Abuse: A Journal of Research and Treatment*, *24*(5), 479–500. https://doi.org/10.1177/1079063212438922

Carvalho, J., Bradford, J., Murphy, L., Briken, P., & Fedoroff, P. (2020). Measuring Pedophilic Sexual Interest. *The Journal of Sexual Medicine*, *17*(3), 378–392. https://doi.org/10.1016/j.jsxm.2019.12.008

Chivers, M. L., Seto, M. C., Lalumière, M. L., Laan, E., & Grimbos, T. (2010). Agreement of Self-Reported and Genital Measures of Sexual Arousal in Men and Women: A Meta-Analysis. *Archives of Sexual Behavior*, *39*(1), 5–56. https://doi.org/10.1007/s10508-009-9556-9

Crooks, V. L., Rostill-Brookes, H., Beech, A. R., & Bickley, J. A. (2009). Applying Rapid Serial Visual Presentation to Adolescent Sexual Offenders : Attentional bias as a measure of deviant sexual interest? *Sexual Abuse: A Journal of Research and Treatment*, *21*(2), 135–148. https://doi.org/10.1177/1079063208328677

Dawson, D., Barnes-Holmes, D., Gresswell, D. M., Hart, A. J., & Gore, N. J. (2009). Assessing the Implicit Beliefs of Sexual Offenders Using the Implicit Relational Assessment Procedure. *Sexual Abuse: A Journal of Research and Treatment*, *21*(1), 57–75. https://doi.org/10.1177/1079063208326928

Dombert, B., Antfolk, J., Kallvik, L., Zappalà, A., Osterheider, M., Mokros, A., & Santtila, P. (2015). Identifying Pedophilic Interest in Sex Offenders Against Children With the Indirect Choice Reaction Time Task. *European Journal of Psychological Assessment*, *33*(5), 1–7. https://doi.org/10.1027/1015-5759/a000293

Flak, V. E., Beech, A. R., & Humphreys, G. W. (2009). The Rapid Serial Visual Presentation Test of Sexual Interest in Child Molesters. *Cognitive Approaches to the Assessment of Sexual Interest in Sexual Offenders*, *DECEMBER 2009*, 145–158. https://doi.org/10.1002/9780470747551.ch7

Fromberger, P., Jordan, K., Steinkrauss, H., von Herder, J., Stolpmann, G., Kröner-Herwig, B., & Müller, J. L. (2013). Eye movements in pedophiles: Automatic and controlled attentional processes while viewing prepubescent stimuli. *Journal of Abnormal Psychology*, *122*(2), 587–599. https://doi.org/10.1037/a0030659

Fromberger, P., Jordan, K., Steinkrauss, H., von Herder, J., Witzel, J., Stolpmann, G., Kröner-Herwig, B., Müller, J. L., Kröner‐Herwig, B., & Müller, J. L. (2012). Diagnostic Accuracy of Eye Movements in Assessing Pedophilia. *Journal of Sexual Medicine*, *9*(7), 1868–1882. https://doi.org/10.1111/j.1743-6109.2012.02754.x

Fromberger, P., Jordan, K., von Herder, J., Steinkrauss, H., Nemetschek, R., Stolpmann, G., & Müller, J. L. (2012). Initial Orienting Towards Sexually Relevant Stimuli: Preliminary Evidence from Eye Movement Measures. *Archives of Sexual Behavior*, *41*(4), 919–928. https://doi.org/10.1007/s10508-011-9816-3

Gray, N. S., Brown, A. S., MacCulloch, M. J., Smith, J., & Snowden, R. J. (2005). An Implicit Test of the Associations Between Children and Sex in Pedophiles. *Journal of Abnormal Psychology*, *114*(2), 304–308. https://doi.org/10.1037/0021-843X.114.2.304

Gress, C. L. Z., & Laws, D. R. (2009). Cognitive Modelling of Sexual Arousal and Interest: Choice Reaction Time Measures. In *Cognitive Approaches to the Assessment of Sexual Interest in Sexual Offenders* (pp. 85–99). Wiley-Blackwell. https://doi.org/10.1002/9780470747551.ch4

Hempel, I. S., Buck, N. M. L., Goethals, K. R., & van Marle, H. J. C. (2013). Unraveling Sexual Associations in Contact and Noncontact Child Sex Offenders Using the Single Category – Implicit Association Test. *Sexual Abuse: A Journal of Research and Treatment*, *25*(5), 444–460. https://doi.org/10.1177/1079063212464660

Hu, S.-H., Wang, Q.-D., Xu, Y., Liao, Z.-L., Xu, L.-J., Liao, Z.-L., Xu, X.-J., Wei, E.-Q., Yan, L.-Q., Hu, J.-B., Wei, N., Zhou, W.-H., Huang, M.-L., & Zhang, M.-M. (2011). Haemodynamic Brain Response to Visual Sexual Stimuli is Different between Homosexual and Heterosexual Men. *Journal of International Medical Research*, *39*(1), 199–211. https://doi.org/10.1177/147323001103900121

Huberman, J. S., & Chivers, M. L. (2015). Examining gender specificity of sexual response with concurrent thermography and plethysmography. *Psychophysiology*, *52*(10), 1382–1395. https://doi.org/10.1111/psyp.12466

Jones, S. C. (2013). The impact of sexual arousal on the category specificity of women’s visual attention to erotic stimuli. In *ProQuest Dissertations and Theses*. http://ezproxy.net.ucf.edu/login?url=http://search.proquest.com/docview/1466645945?accountid=10003%5Cnhttp://sfx.fcla.edu/ucf?url_ver=Z39.88-2004&rft_val_fmt=info:ofi/fmt:kev:mtx:dissertation&genre=dissertations+&+theses&sid=ProQ:ProQuest+Dissertations+&+

Jordan, K., Fromberger, P., von Herder, J., Steinkrauss, H., Nemetschek, R., Witzel, J., & Müller, J. L. (2016). Impaired Attentional Control in Pedophiles in a Sexual Distractor Task. *Frontiers in Psychiatry*, *7*(December), 193. https://doi.org/10.3389/fpsyt.2016.00193

Jordan, K., Jordan, K., Fromberger, P., & Müller, J. L. (2015). *Could we measure sexual interest using functional imaging ? Could we measure sexual interest using functional imaging ?* *AUGUST*, 1–29.

Kukkonen, T. M., Binik, Y. M., Amsel, R., & Carrier, S. (2007). Thermography as a Physiological Measure of Sexual Arousal in Both Men and Women. *The Journal of Sexual Medicine*, *4*(1), 93–105. https://doi.org/10.1111/j.1743-6109.2006.00399.x

Kukkonen, T. M., Binik, Y. M., Amsel, R., & Carrier, S. (2010). An Evaluation of the Validity of Thermography as a Physiological Measure of Sexual Arousal in a Non-University Adult Sample. *Archives of Sexual Behavior*, *39*(4), 861–873. https://doi.org/10.1007/s10508-009-9496-4

Mitricheva, E., Kimura, R., Logothetis, N. K., & Noori, H. R. (2019). Neural substrates of sexual arousal are not sex dependent. *Proceedings of the National Academy of Sciences*, *116*(31), 15671–15676. https://doi.org/10.1073/pnas.1904975116

Ó Ciardha, C., & Gormley, M. (2012). Using a Pictorial-Modified Stroop Task to Explore the Sexual Interests of Sexual Offenders Against Children. *Sexual Abuse: A Journal of Research and Treatment*, *24*(2), 175–197. https://doi.org/10.1177/1079063211407079

Ó Ciardha, Caoilte, Attard-Johnson, J., & Bindemann, M. (2018). Latency-Based and Psychophysiological Measures of Sexual Interest Show Convergent and Concurrent Validity. *Archives of Sexual Behavior*, *47*(3), 637–649. https://doi.org/10.1007/s10508-017-1133-z

Poeppl, T. B., Langguth, B., Rupprecht, R., Laird, A. R., & Eickhoff, S. B. (2016). A neural circuit encoding sexual preference in humans. *Neuroscience & Biobehavioral Reviews*, *68*, 530–536. https://doi.org/10.1016/j.neubiorev.2016.06.025

Poeppl, T. B., Nitschke, J., Santtila, P., Schecklmann, M., Langguth, B., Greenlee, M. W., Osterheider, M., & Mokros, A. (2013). Association between brain structure and phenotypic characteristics in pedophilia. *Journal of Psychiatric Research*, *47*(5), 678–685. https://doi.org/10.1016/j.jpsychires.2013.01.003

Ponseti, J. (2012a). Assessment of Pedophilia Using Hemodynamic Brain Response to Sexual Stimuli. *Archives of General Psychiatry*, *69*(2), 187. https://doi.org/10.1001/archgenpsychiatry.2011.130

Ponseti, J. (2012b). Assessment of Pedophilia Using Hemodynamic Brain Response to Sexual Stimuli. *Archives of General Psychiatry*, *69*(2), 187. https://doi.org/10.1001/archgenpsychiatry.2011.130

Ponseti, J., Bruhn, D., Nolting, J., Gerwinn, H., Pohl, A., Stirn, A., Granert, O., Laufs, H., Deuschl, G., Wolff, S., Jansen, O., Siebner, H., Briken, P., Mohnke, S., Amelung, T., Kneer, J., Schiffer, B., Walter, H., & Kruger, T. H. C. (2018). Decoding Pedophilia: Increased Anterior Insula Response to Infant Animal Pictures. *Frontiers in Human Neuroscience*, *11*. https://doi.org/10.3389/fnhum.2017.00645

Ponseti, J., Siebner, H. R., Klöppel, S., Wolff, S., Granert, O., Jansen, O., Mehdorn, H. M., & Bosinski, H. A. (2007). Homosexual women have less grey matter in perirhinal cortex than heterosexual women. *PLoS ONE*, *2*(8), e762. https://doi.org/10.1371/journal.pone.0000762

Rieger, G., Cash, B. M., Merrill, S. M., Jones-Rounds, J., Dharmavaram, S. M., & Savin-Williams, R. C. (2015). Sexual arousal: The correspondence of eyes and genitals. *Biological Psychology*, *104*, 56–64. https://doi.org/10.1016/j.biopsycho.2014.11.009

Rieger, G., & Savin-Williams, R. C. (2012). The Eyes Have It: Sex and Sexual Orientation Differences in Pupil Dilation Patterns. *PLoS ONE*, *7*(8), e40256. https://doi.org/10.1371/journal.pone.0040256

Rönspies, J., Schmidt, A. F., Melnikova, A., Krumova, R., Zolfagari, A., & Banse, R. (2015). Indirect Measurement of Sexual Orientation: Comparison of the Implicit Relational Assessment Procedure, Viewing Time, and Choice Reaction Time Tasks. *Archives of Sexual Behavior*, *44*(5), 1483–1492. https://doi.org/10.1007/s10508-014-0473-1

Smith, P., & Waterman, M. (2003). Processing bias for aggression words in forensic and nonforensic samples. *Cognition & Emotion*, *17*(5), 681–701. https://doi.org/10.1080/02699930302281

Snowden, R. J., & Gray, N. S. (2013). Implicit Sexual Associations in Heterosexual and Homosexual Women and Men. *Archives of Sexual Behavior*, *42*(3), 475–485. https://doi.org/10.1007/s10508-012-9920-z

Snowden, R. J., McKinnon, A., Fitoussi, J., & Gray, N. S. (2019). Pupillary Responses to Static Images of Men and Women: A Possible Measure of Sexual Interest? *The Journal of Sex Research*, *56*(1), 74–84. https://doi.org/10.1080/00224499.2017.1394959

Spada, A. H., & Jeglic, E. L. (2016). A cognitive-based indicator of deviant sexual interest: Concurrent validation of the Stroop task. *Journal of Sexual Aggression*, *22*(2), 246–262. https://doi.org/10.1080/13552600.2015.1078000

Strahler, J., Baranowski, A. M., Walter, B., Huebner, N., & Stark, R. (2019). Attentional bias toward and distractibility by sexual cues: A meta-analytic integration. *Neuroscience and Biobehavioral Reviews*, *105*(August 2018), 276–287. https://doi.org/10.1016/j.neubiorev.2019.07.015

Tavares, I. M., Vardasca, R., Cera, N., Pereira, R., Nimbi, F. M., Lisy, D., Janssen, E., & Nobre, P. J. (2018). A review of infrared thermography as applied to human sexual psychophysiology. *International Journal of Psychophysiology*, *133*, 28–40. https://doi.org/10.1016/j.ijpsycho.2018.09.001

Vásquez-Amézquita, M., Leongómez, J. D., Seto, M. C., Bonilla, M., Rodríguez-Padilla, A., & Salvador, A. (2019). Visual Attention Patterns Differ in Gynephilic and Androphilic Men and Women Depending on Age and Gender of Targets. *The Journal of Sex Research*, *56*(1), 85–101. https://doi.org/10.1080/00224499.2017.1372353

Wenzlaff, F., Briken, P., & Dekker, A. (2016). Video-Based Eye Tracking in Sex Research: A Systematic Literature Review. *The Journal of Sex Research*, *53*(8), 1008–1019. https://doi.org/10.1080/00224499.2015.1107524

Zappalà, A., Antfolk, J., Bäckström, A., Dombert, B., Mokros, A., & Santtila, P. (2013). Differentiating sexual preference in men: Using dual task rapid serial visual presentation task. *Scandinavian Journal of Psychology*, *54*(4), 320–327. https://doi.org/10.1111/sjop.12050

Zappalà, A., Antfolk, J., Dombert, B., Mokros, A., & Santtila, P. (2016). Identifying deviant sexual interest in a sex offender sample using dual-target rapid serial visual presentation task. *The Journal of Forensic Psychiatry & Psychology*, *27*(2), 281–307. https://doi.org/10.1080/14789949.2015.1122820
